# Eugenol Inhibits Neutrophils Myeloperoxidase In Vitro and Attenuates LPS-Induced Lung Inflammation in Mice

**DOI:** 10.3390/ph17040504

**Published:** 2024-04-15

**Authors:** Amina Chniguir, Mohamed Hedi Saguem, Pham My-Chan Dang, Jamel El-Benna, Rafik Bachoual

**Affiliations:** 1 Faculty of Sciences of Gabes, University of Gabes, Gabes 6029, Tunisia; chniguiramina@gmail.com; 2 Laboratory of Anatomy and Pathological Cytology, Gabes 6029, Tunisia; mh.saguem@topnet.tn; 3 INSERM U1149, CNRS ERL8252 Inflammation Research Center, 75018 Paris, France; my-chan.dang@inserm.fr (P.M.-C.D.); jamel.elbenna@inserm.fr (J.E.-B.); 4 Inflamex Laboratories, Faculty of Medicine, University of Paris City, Xavier Bichat, 75018 Paris, France

**Keywords:** eugenol, neutrophils, oxidative stress, lung inflammation, myeloperoxidase, metalloproteinases

## Abstract

Eugenol (Eug) is a polyphenol extracted from the essential oil of *Syzygium aromaticum* (L.) Merr. and Perry (Myrtaceae). The health benefits of eugenol in human diseases were proved in several studies. This work aims to evaluate the effect of eugenol on lung inflammatory disorders. For this, using human neutrophils, the antioxidant activity of eugenol was investigated in vitro. Furthermore, a model of LPS-induced lung injury in mice was used to study the anti-inflammatory effect of eugenol in vivo. Results showed that eugenol inhibits luminol-amplified chemiluminescence of resting neutrophils and after stimulation with N-formyl-methionyl-leucyl-phenylalanine (fMLF) peptide or phorbol myristate acetate (PMA). This effect was dose dependent and was significant from a low concentration of 0.1 µg/mL. Furthermore, eugenol inhibited myeloperoxidase (MPO) activity without affecting its degranulation. Eugenol has no scavenging effect on hydrogen peroxide (H_2_O_2_) and superoxide anion (O_2_^−^). Pretreatment of mice with eugenol prior to the administration of intra-tracheal LPS significantly reduced neutrophil accumulation in the bronchoalveolar lavage fluid (BALF) and decreased total proteins concentration. Moreover, eugenol clearly inhibited the activity of matrix metalloproteinases MMP-2 (21%) and MMP-9 (28%), stimulated by LPS administration. These results suggest that the anti-inflammatory effect of eugenol against the LPS-induced lung inflammation could be exerted via inhibiting myeloperoxidase and metalloproteinases activity. Thus, eugenol could be a promising molecule for the treatment of lung inflammatory diseases.

## 1. Introduction

Chronic inflammation and oxidative stress are major causes for the development of different pathologies like cardiovascular diseases, rheumatoid arthritis, cancer, and respiratory diseases [1]. The pathogenesis of these diseases involves the major cells of the immune system including macrophages, neutrophils, basophils, T-cells, and lymphocytes [2]. Activated neutrophils produce increased levels of reactive oxygen species (ROS), release a multitude of enzymes and proteins such as myeloperoxidase, proteases, lysozyme, lactoferrin, and lipocalin implicated in microbe killing and inflammatory reactions. Pro-inflammatory mediators such as cytokines (IL-1, IL-6, TNF, etc.), ROS, and proteolytic enzymes are implicated in cell and tissue damages associated with many chronic inflammatory diseases, including chronic obstructive pulmonary disease (COPD) [3]. Cigarette smoke is the most known risk factor for the development of COPD, but it can also be induced by other harmful particles [4]. COPD is characterized by an increase in inflammatory cells number, high levels of inflammatory mediators, and an imbalance in oxidant/antioxidant and proteases/antiproteases production, leading to an irreversible airflow obstruction and mucus accumulation [2]. COPD is a major cause of morbidity and is the fourth leading cause of death worldwide [5]. Due to its physio-pathological complexity, no curative molecules are available to treat this disease except some steroidal anti-inflammatory drugs with known side effects. Moreover, the effects of the available therapies are limited to minimizing symptoms, reducing exacerbation risk, and improving quality of life of patients [6]. Thus, the identification of novel treatments for this disease requires exploring new active molecule candidates that can target inflammation and mucus accumulation. Historically, the plant kingdom is the main source of natural products with a vast range of biological activities [7]. Polyphenols are one of the most numerous and ubiquitous groups of plant metabolites, and their antioxidant and anti-inflammatory potential have been widely recognized [8]. Eugenol (4-allyl-2-methoxyphenol) is a polyphenol identified in the composition of essential oils of many plants, but it is known as the main compound derived from *Syzygium aromaticum* (L.) Merr. and Perry (Myrtaceae), a spice widely used in the diets of many populations in the world [9]. Previous studies reported that eugenol is a topical local anesthetic and it has potent biological activities. It has been observed that eugenol possesses antimicrobial, anti-tumor, antioxidant, and anti-inflammatory effects [10]. Pharmacokinetic and metabolic studies demonstrate that eugenol exerts its biological effects via its antioxidant proprieties. In a previous in vitro study, our team demonstrated that eugenol significantly decreased the production of superoxide anion by fMLF-stimulated human neutrophils via the inhibition of NADPH oxidase phosphorylation and activation [11]. Furthermore, a previous study reported the inhibitory effect of eugenol on lipid peroxidation [12]. Hence, the present work aimed to examine the effect of eugenol on LPS-induced lung inflammation in mice and study its antioxidant effects in vitro.

## 2. Results

### 2.1. Eugenol Significantly Decreases ROS Production in Human Neutrophils

**Figure 1:**
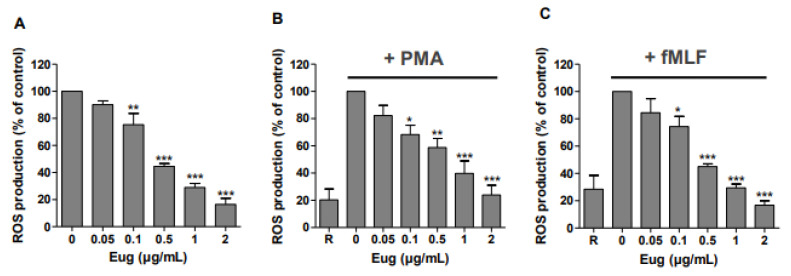
Eugenol inhibits dose dependently ROS production in human neutrophils. Neutrophils (5 × 10^5^) were incubated in the absence or presence of increasing concentrations of eugenol for 30 min with 10 µM of luminol. ROS production was measured in resting neutrophils (R: resting cells) (**A**) and after stimulation with PMA (100 ng/mL) (**B**) of fMLF (10^−6^ M) (**C**) using luminol-enhanced chemiluminescence assay during 30 min. Results were expressed as a percentage to control (mean ± SEM from three or more separate experiments, * *p* < 0.05, ** *p* < 0.005, *** *p* < 0.001).

To evaluate the antioxidant effect of eugenol, ROS production by human neutrophils was analyzed using luminol-amplified chemiluminescence. Results showed that eugenol dose-dependently (0.05–2 µg/mL) inhibited the production of reactive oxygen species (ROS) by human neutrophils in resting conditions (Figure 1A) and after their stimulation with two different agonists PMA (Figure 1B) or fMLF (Figure 1C). The inhibitory effect was significant from low concentration of 0.1 µg/mL with IC_50_ value of 0.5 µg/mL.

### 2.2. Eugenol Does Not Scavenge Superoxide Anions nor Hydrogen Peroxide

**Figure 2:**
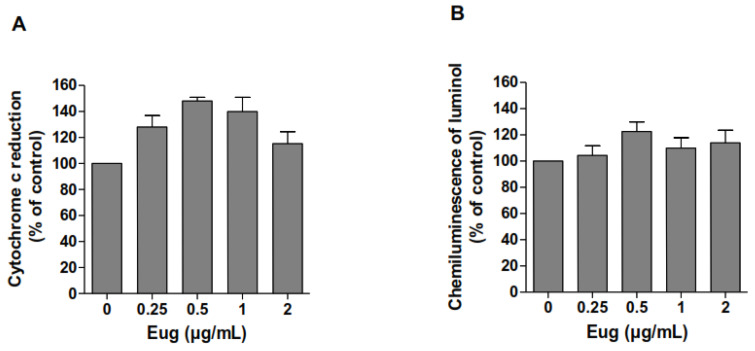
Eugenol does not scavenge O_2_^−^ (**A**) or H_2_O_2_ (**B**). Scavenging effect of eugenol was examined in free-cell systems using xanthine/xanthine oxidase and H_2_O_2_/HRPO, respectively. Results were expressed as a percentage to control (mean ± SEM of three or more separate experiments, *p* < 0.05).

To study the scavenging effect of eugenol against superoxide anions and hydrogen peroxide, cell-free systems were used. The direct effect of eugenol on superoxide anion and hydrogen peroxide was studied using xanthine/xanthine oxidase and H_2_O_2_/HRPO systems, respectively. Results showed that eugenol had no scavenging effect on O_2_^−^ (Figure 2A) or H_2_O_2_ (Figure 2B). Taken together, our findings suggest that the effect of eugenol could probably be explained by direct effect on the enzymes responsible for ROS generation, the NADPH oxidase, or the myeloperoxidase.

### 2.3. Eugenol Inhibits Strongly Myeloperoxidase Activity

**Figure 3:**
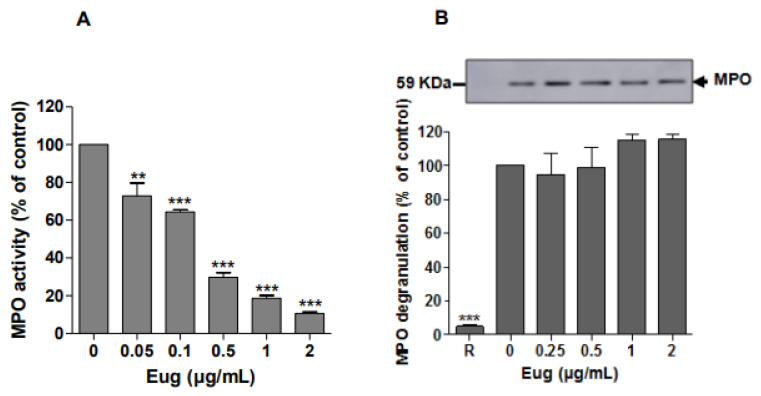
Eugenol strongly inhibits MPO activity without affecting its cellular release. MPO was incubated in the absence or presence of increasing concentrations of eugenol, and its activity was measured using tetra-methylbenzidine (TMB) oxidation assay at 650 nm (**A**). Human neutrophils (10 × 10^6^ cells) were incubated in the absence or presence of eugenol for 30 min at 37 °C. Release of MPO from azurophilic granules was stimulated by 10^−6^ M of fMLF for 15 s in the presence of 5 µg/mL of cytochalasin B. Stimulation was stopped by a 30 s centrifugation at 13,000 rpm. MPO in the supernatant was detected by Western blotting and quantified using Image J 1.54d software (**B**). Results were expressed as a percentage to control (mean ± SEM of three or more separate experiments, ** *p* < 0.005, *** *p* < 0.001). (R: resting cells).

Myeloperoxidase (MPO) is an important member of innate immune defense and oxygen-dependent microbicidal activity of phagocytes [13]. It is released from the azurophilic granules of activated neutrophils and generates important amounts of hypochloric acid (HOCl), a major ROS molecule. Excessive activity of MPO leads to the initiation and progression of various inflammatory diseases [14]. Thus, we therefore tested the effect of eugenol on MPO activity and its release from neutrophils azurophilic granules. As shown in Figure 3A, eugenol significantly inhibits (*p* < 0.01) the activity of MPO in a dose-dependent manner from a low concentration of 0.05 µg/mL without affecting its discharge from fMLF-stimulated neutrophils (Figure 3B).

### 2.4. Eugenol Attenuates the LPS-Induced Inflammatory Response in Mice

**Figure 4:**
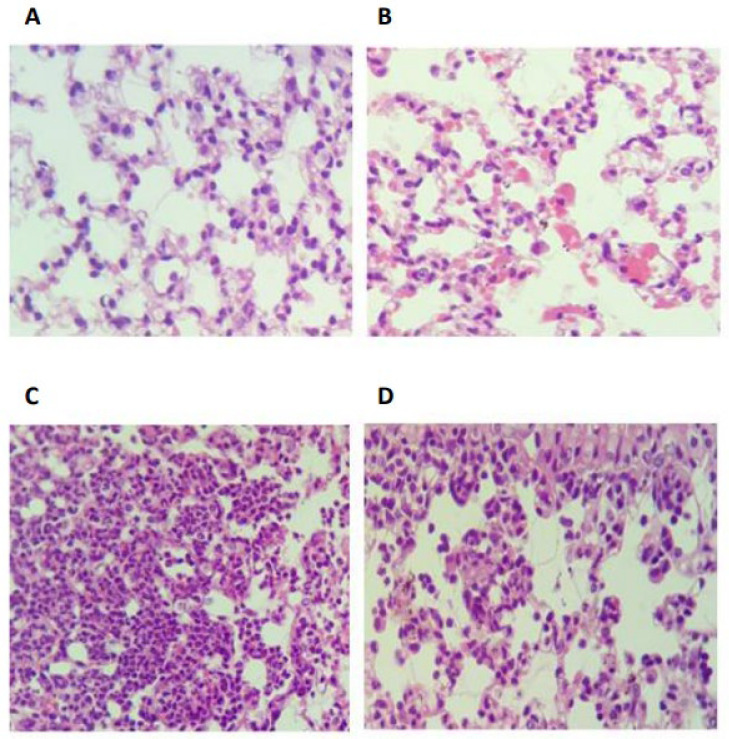
Effect of eugenol on lung histology (magnification 200×). (**A**) Control saline, (**B**) eugenol (200 mg/kg), (**C**) LPS (5 µg/mouse), (**D**) eugenol + LPS.

**Figure 5:**
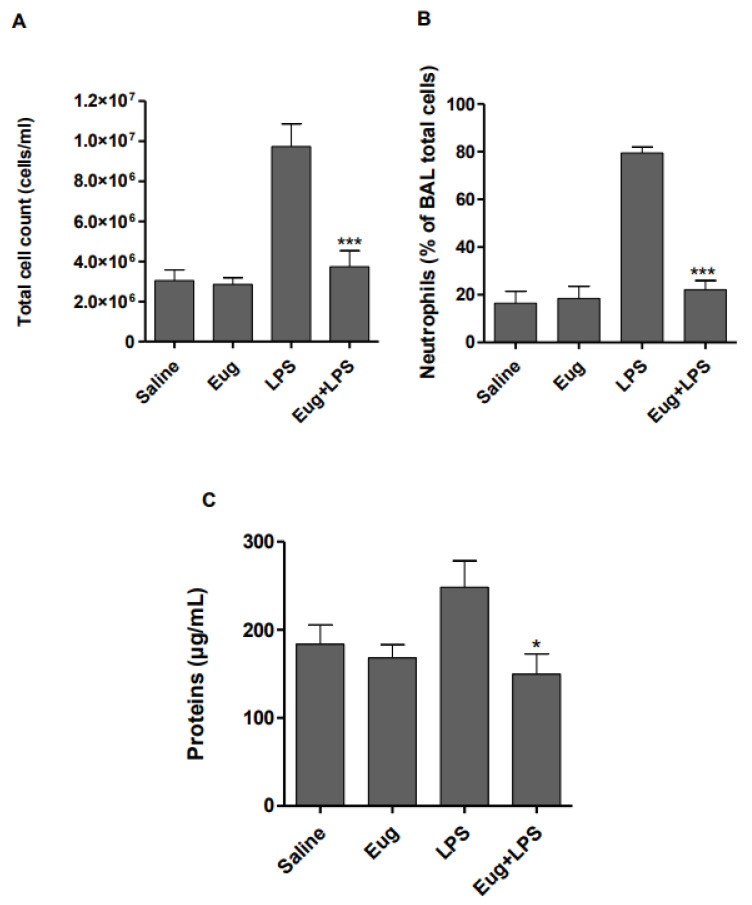
Eugenol decreased total and differential cell count and inhibited neutrophils infiltration in the BALF. Swiss mice were treated or not intraperitoneally with eugenol (200 mg/Kg) prior to the induction of lung inflammation by intra-tracheal instillation of LPS (5 µg/mouse). The BALF was collected, and the total cell count (**A**) and neutrophil number (**B**) were determined by light microscopy. Total protein concentrations in the BALF were evaluated using Quick-Start Bradford assay (**C**). The results are expressed as means of 8 mice (mean ± SEM, * *p* < 0.05, *** *p* < 0.001).

Intra-tracheal instillation of LPS is a common experimental model used to investigate the molecular mechanisms and drug’s efficacy in lung inflammation studies. To examine the effect of eugenol on LPS-induced lung injury in mice, the morphological features of lungs from different groups were evaluated by light microscopy. Compared to saline (Figure 4A) and eugenol (Figure 4B), LPS induced several histological changes including marked infiltration of inflammatory cells into the alveolar space (Figure 4C). These pathologic symptoms were dramatically reduced following pretreatment with eugenol (Figure 4D). To assess the alveolar capillary permeability, the total protein concentration in BALF was determined. Results (Figure 5C) showed that LPS challenge caused a significant increase of total protein concentration compared with the saline control group. In contrast, injection of eugenol clearly decreases the levels of total proteins concentrations (*p* < 0.5). It was found, also, that the administration of LPS increased the number of BALF inflammatory cells threefold compared with the saline-instilled group. However, the pretreatment of mice with eugenol (200 mg/kg) significantly decreased (*p* < 0.001) inflammatory cell count in BALF (Figure 5A). The number of neutrophils was decreased from 79% to 22% (Figure 5B).

### 2.5. Eugenol Reduces MMP-2 and MMP-9 Activity in Lung Homogenates

**Figure 6:**
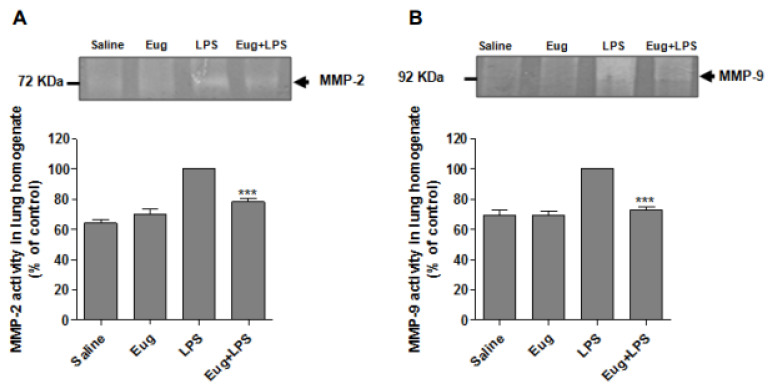
Eugenol decreased matrix metalloproteinases activity in lung homogenates. Lungs from different studied groups were homogenized and centrifuged. MMP-2 (**A**) and (MMP-9) (**B**) activity was detected in the supernatant using gelatin zymography. Results were expressed as a percentage to control (n = 8, mean ± SEM, *** *p* < 0.001).

In order to investigate whether eugenol inhibits metalloproteases activity, gelatin zymography was carried out using the supernatant of lung homogenates from different studied groups. As demonstrated in Figure 6, compared with control group (saline), the enzymatic activity of gelatinases (MMP-2, MMP-9) was dramatically increased in lung homogenates of LPS group. In contrast, significant inhibition (*p* < 0.001) of MMP-2 (21%) (Figure 6A) and MMP-9 (28%) (Figure 6B) activity was observed in the lungs of mice treated with eugenol before LPS instillation.

## 3. Discussion

Eugenol is the major component of *Syzygium aromaticum* essential oil. Several studies demonstrate its health benefits, particularly in inflammatory diseases. In this study, we found that eugenol inhibits the production of reactive oxygen species (ROS) by human neutrophils in resting conditions and after stimulation with two different agonists, PMA or fMLF. The inhibitory effect is significant and dose dependent from the low concentration of 0.1 µg/mL. In addition, eugenol clearly inhibited MPO activity at the low dose of 0.05 µg/mL. In contrast, we showed that eugenol could not affect the degranulation of neutrophils and has no scavenging effect of H_2_O_2_ and O_2_^−^. An in vivo study showed that eugenol inhibited LPS-induced lung inflammation in mice and reduced the activity of matrix metalloproteases MMP-2 and MMP-9 in lung homogenates.

In chronic obstructive pulmonary disease (COPD), one of the lung inflammatory disorders, the oxidative stress acts as the basic etiology [15]. Inflammatory cells, especially neutrophils, recruited in the site of inflammation, generate a number of reactive oxygen species (ROS) via the activation of NOX2 [16]. These ROS damage the surrounding cells, leading to the development of inflammatory disorders [17]. Thus, pharmacological inhibition of ROS overproduction can restrict inflammatory responses [18]. In this study, luminol-enhanced chemiluminescence assays indicated that eugenol significantly decreased total (intracellular and extracellular) ROS generation induced by the protein kinase C activator, PMA, or the chemotactic peptide fMLF. PMA and fMLF are two known NADPH oxidase activators via different transduction pathways [19]. Thus, the inhibitory effect of eugenol on ROS production suggests that it does not affect a specific pathway, but it inhibits a common target such as NADPH oxidase, MPO, or scavenges reactive oxygen species. Luminol-amplified chemiluminescence is dependent on several elements including superoxide anions, H_2_O_2_, and MPO release and activity [20]. Hence, we found that eugenol does not affect H_2_O_2_/HRPO cell-free chemiluminescence but it strongly inhibits myeloperoxidase activity at low concentration (0.05 µg/mL) without affecting its cellular release. MPO is an important member of innate immune defense and oxygen-dependent microbicidal activity of phagocytes [21]. It is released from the azurophilic granules of activated neutrophils. Excessive release of MPO leads to the initiation and progression of various inflammatory diseases [22]. A recent study showed that the inhibition of MPO activity, using its pharmacological inhibitor AZM198, reduced degranulation of ANCA-stimulated neutrophils and attenuated endothelial cell damage caused by neutrophils infiltration. Moreover, in a model of crescentic glomerulonephritis, MPO inhibition suppressed kidney damage without increase in adaptative T cell responses [23]. Thus, MPO could be a target for the treatment of inflammatory diseases. For this, there is a growing interest in the research and development of MPO inhibitors from natural molecules. A comparative study of the effect of fifteen essential oils on MPO activity in human neutrophils in vitro demonstrated that eugenol exhibits the highest inhibition of MPO activity with IC_50_ of 19.2 µg/mL [24].

LPS is known as a potent inflammatory stimulus used to induce lung inflammation in animal models [25]. It activates the transmembrane receptor TLR-4, which is expressed in pro-inflammatory cells [26]. Activated macrophages induce an influx of inflammatory cells, especially neutrophils, which produce increasing levels of pro-inflammatory cytokines and reactive oxygen species [27]. ROS production leads to the activation of many signaling proteins including NF-KB, MAPKs, iNOS, and COX-2, which accentuate the inflammatory response [28]. Recruitment of neutrophils is an important step in the development of lung injury. Thus, the inhibition of neutrophils infiltration can be a target to attenuate lung inflammation. Our results showed that eugenol reduces the increased influx of neutrophils into lung parenchyma and BALF. Furthermore, we found that eugenol down regulated total protein levels and decreased the formation of lung edema. Since reactive oxygen species are involved in inducing inflammation, the results of this work can be explained in part by our previous study clearly demonstrating the antioxidant effect of eugenol as a major inhibitor of NADPH oxidase activity by inhibiting the Raf/MEK/ERK1/2/p47phox-phosphorylation pathway [11]. One other study has shown that a specific inhibitor of COX-2 significantly reduced neutrophil recruitment into lung adenocarcinoma A549, which was associated with slower-growing tumors [29]. It was shown also that the neutralization of interleukine-8 (IL-8), a major chemoattractant of neutrophils, significantly reduced neutrophils count into the bronchoalveolar space and protected rabbits from acid-aspiration-induced lung injury [30].

Matrix metalloproteinases (MMPs) are a family of endopeptidases able to degrade the denatured fibrillar collagens, elastase, and several other components of the extracellular matrix. They are produced by different cell types, notably neutrophils [31]. MMPs are implicated in many aspects of both physiological cellular processes and pathological mechanisms [32]. Tissue inhibitors of metalloproteinases (TIMPs) are considered as major endogenous inhibitors of MMPs in physiological conditions [33]. However, in pathological conditions, exogenous MMPs inhibitors may be either synthetic or natural ones and are required to stop the deleterious effect of MMPs. The MMPs family exhibits a structural homology between each other but also to other zinc-dependent proteases [34]. Accordingly, many developed inhibitors fail due the lack of specificity. Thus, there is a need for a high specific inhibitor able to discriminate between the homologous MMPs and ideally administrated as a short-term topical treatment [35]. In this work, we have shown that eugenol can indeed inhibit MMP-2 and MMP-9 activity induced by LPS administration. Indeed, it was already shown that eugenol significantly inhibited the activity of MMP-9, in the concentration range of 10–100 µM, in PMA-stimulated H1080 cells via the reduction of oxidative stress parameters [36]. Furthermore, Abdullah et al. demonstrated that the treatment of breast cell lines MDA-231 and SK-BR-3 with eugenol at 4 µM and 8 µM for 48 h significantly inhibited their proliferation with an inhibition rate of 76.4% and 68.1% respectively. Eugenol-treated cells showed also decreased MMP-2 activity and increased proportion of cells in late apoptosis [37].

Natural bioactive molecules found in medicinal plants are attractive for the development of effective drugs for several diseases especially those associated with inflammatory and oxidant disorders [38]. Epidemiological studies have shown that populations of consumers of fruits and vegetables rich in polyphenols, exhibiting high antioxidant proprieties, have a lower incidence of developing inflammatory diseases [39,40]. As a natural polyphenol, eugenol has shown a beneficial effect in a variety of inflammatory models. In fact, a Brazilian group has shown that the oral administration of eugenol, at doses of 200 mg/kg and 400 mg/Kg, significantly reduced paw edema, 2–4 h after carrageenan injection in mice. The inhibition was comparable to each of indomethacin and celexib, two known anti-inflammatory molecules [41]. Another in vitro study demonstrated that eugenol exhibits a tumor suppressive effect on lung cancer cells. It inhibits cell viability, proliferation, migration, and invasion at all tested concentrations (50 µM to 1000 µM). This effect was explained by the inhibition of PI3K/AKT pathway and the reduction of MMP-2 activity, supporting the use of eugenol as chemotherapeutic agent against human lung cancer [42].

## 4. Materials and Methods

### 4.1. Chemicals and Reagents

Eugenol, *LPS* (from *Escherichia coli* strain O55:B6), luminol, cytochrome c, N-formyl-methionyl-leucyl-phenylalanine (fMLF), and phorbol myristate acetate (PMA) were purchased from Sigma-Aldrich (Saint-Quentin Fallavier, France). Ficoll and Dextran T500 were purchased from GE Healthcare.

### 4.2. Preparation of Eugenol

Pure eugenol (purity: 99%) was dissolved in DMSO (dimethylsulfoxide) to maximize its solubility, and different concentrations used for the in vitro study were prepared in Hank’s Balanced Salt Solution (HBSS). A concentration range from 0.05 µg/mL to 2 µg/mL was tested, correspending to 12 µM as maximal concentation. As for the in vivo study, the dose of 200 mg/kg of eugenol was chosen, referring to other work which used doses ranging from 200 to 400 mg/kg [41].

### 4.3. Human Neutrophils Isolation

Venous blood was obtained from healthy volunteers after written informed consent had been obtained. The study was approved by the institutional review boards (IRBs) and ethics committee of INSERM (Etablissement Français du Sang (EFS) agreement n°2018010827 (1 January 2018)). All these procedures were conducted in accordance with the 1975 Declaration of Helsinki, as revised in 2013. Data collection and analyses were performed anonymously. Neutrophils were freshly isolated by dextran sedimentation and centrifugation in a Ficoll Hypaque gradient [43]. Red blood cells were removed by hypotonic lysis. Following isolation, the cells were kept in Hank’s Balanced Salt Solution (HBSS) at 4 °C until use.

### 4.4. Luminol-Amplified Chemiluminescence by Human Neutrophils

Neutrophils (5 × 10^5^) were incubated in 0.5 mL of HBSS in the absence or presence of eugenol at a concentration range from 0.05 to 2 µg/mL and luminol (10 µM), preheated at 37 °C in the thermostated chamber of a luminometer (Berthold-Biolumat LB937, France) and allowed to stabilize. Cells were then stimulated by the addition of fMLF (10^−6^ M) or PMA (100 ng/mL). Changes in chemiluminescence were measured over a 30 min period.

### 4.5. Xanthine/Xanthine Oxidase Activity Assay

Xanthine oxidase (5 U.I) was incubated in 0.5 mL of PBS in the absence or presence of eugenol at different concentrations (0.05 to 2 µg/mL) and cytochrome c (1 mg/mL), preheated at 37 °C in the thermostated chamber of a spectrophotometer (Agilent-Technologies, Santa Clara, CA, USA) and allowed to stabilize. The reaction was initiated by the addition of xanthine (100 µM) and changes in optical density (OD) were measured at 550 nm over a 10 min period.

### 4.6. Measurement of H_2_O_2_ Scavenging Effect

Eugenol was preincubated with hydrogen peroxide (80 µM) in the presence of luminol (10 µM) for 15 min in the thermostated chamber of a luminometer (Berthold-Biolumat LB937) and allowed to stabilize. The reactions were initiated by adding HRP (5 U.I.), and changes in chemiluminescence were measured over a 30 min period.

### 4.7. Measurement of Neutrophils MPO Activity

MPO was extracted from sonicated granules of human neutrophils in 0.2% cetyltrimethylammonium (CTAB) [44]. The enzymatic activity was assessed by measuring the H_2_O_2_-dependant oxidation of tetramethyl-benzidine (TMB), at 650 nm.

### 4.8. Neutrophils Degranulation

Neutrophils (5 × 10^6^) were incubated in the absence or presence of increasing concentrations of eugenol for 30 min at 37 °C before the addition of cytochalasin B (5 µg/mL) for 5 min. Then, neutrophils were stimulated with fMLF (10^−6^ M) for 15 s. Stimulation was stopped by immediate centrifugation. Cell-free supernatants were denaturized with Laemli buffer and stored at −80 °C. Samples were subjected to electrophoresis using 10% SDS-PAGE. Subsequently, the separated proteins were transferred to a nitrocellulose membrane. MPO was detected by specific antibody anti-MPO (1:5000). The bands were quantified using the Image J analysis program (National Institute of Health, Bethesda, MD, USA).

### 4.9. Animals

In this research, male Swiss mice in the weight range of 20 to 25 g were bought from Pasteur Institute (Tunis, Tunisia). They were housed in standard wire-topped cages and controlled temperature units. Food and water were supplied ad libitum. The experiments were approved by the Institutional Committee on Animal Care and used at Faculty of sciences of Gabes. All animal experiment protocols complied with ARRIVE guidelines.

### 4.10. Model of Lung Inflammation

To study the effect of eugenol on LPS-induced lung injury, mice were randomly designated into 4 groups, with 8–10 mice in each group. Group (1) [Saline] was control, received saline (NaCl 0.9%) by intraperitoneal (IP) and intra-tracheal (IT) routes. Group (2) [Eug] received 200 mg/kg of eugenol intraperitoneally (IP) and saline by IT route. The third group (3) [LPS] received saline by IP route and LPS (5 µg/mouse) by intra-tracheal instillation. Group (4) [Eug+LPS] was treated intraperitoneally with 200 mg/kg of eugenol and instilled by LPS. Mice received saline (groups 1 and 3) or eugenol (group 2 and 4) intraperitoneally (IP) once per day for two consecutive days. For each group, three hours after the second injection, mice were anesthetized with a cocktail of anesthetics (75 mg/kg ketamine (Virbac Sante Animale, Carros, France) plus 1 mg/kg medetomidine (Pfizer, Paris, France)). Subsequently, they were instilled, intra-tracheally, with 5 µg of LPS (groups 3 and 4) or physiological saline (groups 1 and 2). The mice were aroused by an IP injection of 1 mg/kg atipamezole (Pfizer), a medetomidine antagonist.

### 4.11. BALF Collection and Cell Counting

Animals were anesthetized by IP injection of 50 mg of Pentothal (Sigma, Kanagawa, Japan), lungs were lavaged twice with 1 mL ice cold physiological saline through a tracheal cannula, removed rapidly and immediately frozen in liquid nitrogen and stored at −80 °C for subsequent use. BALF from each sample was centrifuged (4 °C, 3000 rpm, 10 min). The supernatants were stored at −80 °C to determine the total protein concentration with Quick-Start Bradford assay (Bio-Rad, Marnesla-Coquette, France) and to assess metalloproteinases (MMPs) activity by zymography technique. Erythrocytes were removed from the cell pellet by hypotonic lysis followed by two washes with cold sterile saline. The cells were resuspended in 150 µL of physiological saline and an aliquot was used to evaluate total white cell count with a hemocytometer. For differential cellularity, the cell suspension was cytospun (Cytospin-2, Shandon Products Ltd., Levallois-Perret, France), fixed in methanol, and stained with Diff-Quick solution (Medion Diagnostics, Plaisir, France). Three hundred cells were counted with an oil immersion lens (1000×).

### 4.12. Histological Study

The whole lungs were fixed in 10% formalin for 24 h, embedded in paraffin, and sectioned on a microtome. The obtained sections were stained with hematoxylin and eosin, then observed by light microscopy (Magnification 200×) to examine neutrophils infiltration in the lung.

### 4.13. Gelatin Zymography

Metalloproteinases, MMP-2 and MMP-9, activity in lung was assessed using gelatin zymography as previously described [45]. For this, lung tissue was ground, and 10 µg of samples were electrophoresed using 10% SDS-PAGE containing 0.1% gelatin. Gels were rinsed twice with 2.5% Triton X-100 for 30 min at room temperature, subsequently incubated overnight at 37 °C in activation buffer (50 mM Tris-HCL (pH 7), 5 mM CaCl_2_, 1 μM ZnCl_2_, 0.05% NaN_3_). The gels were stained with Coomassie brilliant blue R-250 (Sigma-Aldrich). MMP-2 and MMP-9 activities were evaluated by incubation of the gels in acetic acid 45%, methanol 10%, and H_2_O buffer. Image J software was used for quantification.

### 4.14. Statistical Analysis

Data are expressed as mean ±SEM for at least 3 independent experiments using the one-way analysis of variance (ANOVA). The Newman–Keuls multiple comparisons test was used for the statistical analysis using GraphPad Prism 5 software (GraphPad Software, San Diego, CA, USA). *p* values < 0.05 were considered to indicate a statistically significant difference.

## 5. Conclusions

The current study shows that eugenol strongly inhibits ROS production by human neutrophils in resting conditions and after stimulation with PMA or fMLF. This effect was associated with clear inhibition of MPO activity. Moreover, eugenol had a protective effect in vivo in a model of LPS-induced lung inflammation in mice by inhibiting neutrophils recruitment in the inflammatory site and by reducing matrix metalloproteinases activity (MMP-2, MMP-9). Taken together, our study suggests that eugenol exerts its anti-inflammatory effect in lungs via the inhibition of MPO and metalloproteases activity. Thus, eugenol could be considered as a promising molecule for lung inflammatory disease treatment.

## Data Availability

All relevant data are within the paper.
